# Domestic and international medical students’ need for mental health services

**DOI:** 10.1192/j.eurpsy.2024.1249

**Published:** 2024-08-27

**Authors:** E. Nikolaev, A. Zakharova, D. Hartfelder, S. Petunova, E. Lazareva, N. Maksimova, N. Grigoreva, E. Litvinova, G. Dulina, E. Vasilieva, M. Alhasan

**Affiliations:** ^1^Department of Psychiatry, Medical Psychology and Neurology; ^2^Department of Social and Clinical Psychology; ^3^Medical Faculty, Ulianov Chuvash State University, Cheboksary, Russian Federation

## Abstract

**Introduction:**

Heavy academic loads imposed on medical students explain why it is so important for a university to pay more attention to the issues of maintaining their students’ mental health.

**Objectives:**

To compare the level of mental health and the need for mental health services in domestic and international medical students

**Methods:**

The survey covered 305 domestic and 241 international university students of the Faculty of Medicine. Their mental health level was measured with the SCL-90R questionnaire, their interest to mental health services - by means of a 5-point questionnaire.

**Results:**

The data achieved by measuring the level of mental health with the SCL-90R revealed that in both groups this level is within standard limits. However, the international students showed a higher level of psychopathological distress reflected by GSI index (χ^2^=2.14; р=.03). Both groups have experienced a visit to a psychiatrist or psychotherapist (12.13% and 8.3% correspondingly). Some of them have undergone treatment in connection with their emotional and behavioral problems (3.28% и 3.73%). Currently, they claim, with the same frequency, that they are in need of a psychiatrist’s or psychotherapist’s help (14.43% и 13.28%). Domestic students, as compared with international students show higher need (χ^2^=24.55; р=.001) for a psychologist’s help (34.75% and 16.18%).With different frequency, 65.15% of the international students and 89.5% of the domestic students consider mental health services as necessary.

**Conclusions:**

When providing medical support to medical students, it is important to take into account their need for mental health services and to keep in mind their different cultural backgrounds.

**Disclosure of Interest:**

None Declared

